# Deep Brain Stimulation in Huntington’s Disease—Preliminary Evidence on Pathophysiology, Efficacy and Safety

**DOI:** 10.3390/brainsci6030038

**Published:** 2016-08-30

**Authors:** Lars Wojtecki, Stefan Jun Groiss, Christian Johannes Hartmann, Saskia Elben, Sonja Omlor, Alfons Schnitzler, Jan Vesper

**Affiliations:** 1 Department of Neurology, Medical Faculty, Heinrich-Heine University Düsseldorf, Moorenstrasse 5, Düsseldorf 40225, Germany; groiss@uni-duesseldorf.de (S.J.G.); christian-hartmann@uni-duesseldorf.de (C.J.H.); saskia.elben@med.uni-duesseldorf.de (S.E.); Sonja.Omlor@uni-duesseldorf.de (S.O.); schnitza@uni-duesseldorf.de (A.S.); 2 Institute of Clinical Neuroscience & Medical Psychology, Medical Faculty, Heinrich-Heine University Düsseldorf, Moorenstrasse 5, Düsseldorf 40225, Germany; 3 Department of Functional Neurosurgery and Stereotaxy, Medical Faculty, Heinrich-Heine University Düsseldorf, Moorenstrasse 5, Düsseldorf 40225, Germany; jan.vesper@med.uni-duesseldorf.de

**Keywords:** chorea, Huntington, deep brain stimulation, DBS, safety pathophysiology, recordings, globus pallidus

## Abstract

Huntington’s disease (HD) is one of the most disabling degenerative movement disorders, as it not only affects the motor system but also leads to cognitive disabilities and psychiatric symptoms. Deep brain stimulation (DBS) of the pallidum is a promising symptomatic treatment targeting the core motor symptom: chorea. This article gives an overview of preliminary evidence on pathophysiology, safety and efficacy of DBS in HD.

## 1. Introduction

In this manuscript, the authors update a recent perspective article on brain stimulation in Huntington’s disease (HD), [1] focusing especially on deep brain stimulation (DBS) and its preliminary evidence on safety and efficacy.

HD is an autosomal dominant inherited neurodegenerative disorder [2]. As a consequence of an expanded CAG repeat in the HD gene motor symptoms, psychiatric symptoms and cognitive decline progressively develop. Even though cellular pathology is evident in the whole body, medium spiny neurons in the circumscribed area of the striatum are considered to selectively degenerate in the course of HD and thereby lead to motor symptoms [3], typically including chorea, dystonia and bradykinesia. Especially, choreatic symptoms commonly occur in early stages of HD [4]. Here, the disinhibition of one basal ganglia network circuit is considered to be pivotal [4]. Degeneration of striatal neurons, which project to the indirect pathway of the basal ganglia circuit, cause decreased basal ganglia output [5] and the aforementioned disinhibition. Furthermore, pathological changes in the direct pathway of the basal ganglia circuit have to be taken into account. Structural alterations in the substantia nigra and the cerebellum could also play a crucial role in dystonic or hypokinetic-rigid symptoms [6,7].

The status quo in HD treatment has offered no approved neuroprotective or causal treatment so far. As a consequence, the therapeutic options for HD rely on symptom treatment, which often is not sufficiently effective or causes side effects.

## 2. Evolution of Deep Brain Stimulation for HD

**Figure 1:**
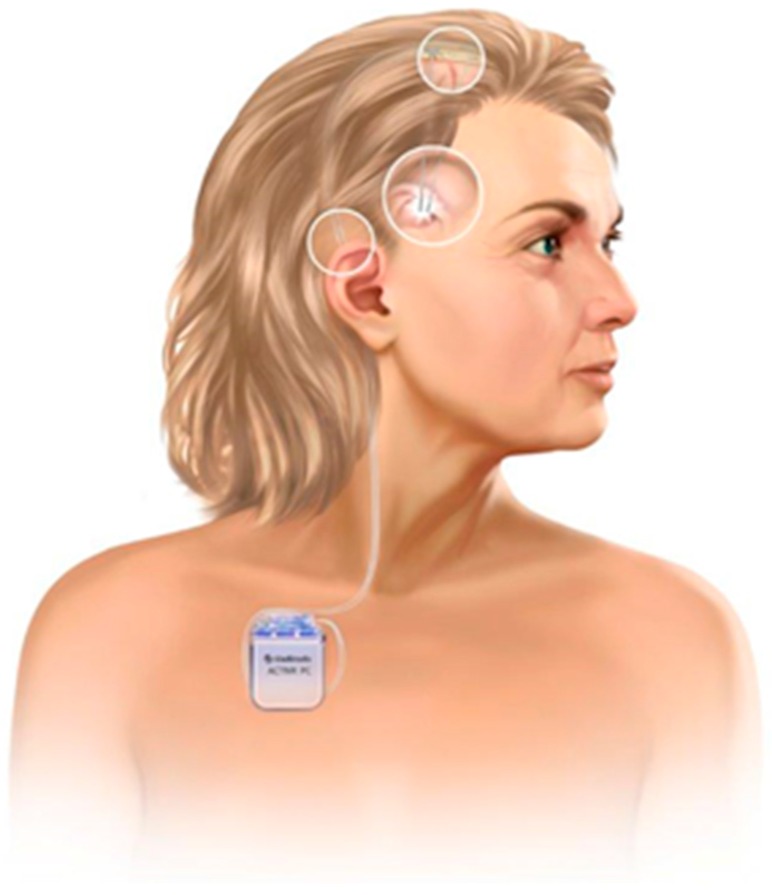
DBS components. Subcutaneous implanted impulse generator (IPG), lead extension and stereotactically implanted stimulation electrodes. Image provided by Medtronic.

Chronic electric stimulation of deep brain structures (see Figure 1) is a well-established therapeutic method using stereotactic techniques to pinpoint the target regions of interest, such as certain parts of the basal ganglia network [8,9]. In 1987, Benabid and colleagues paved the way for the broader DBS application with an implanted impulse generator in different movement disorders and other disorders in the field of neurology and psychiatry [10]. Previous to this, DBS predominantly was (sometimes abusively) proposed for psychiatric disorders [11,12,13]. Subsequent DBS findings in movement disorders such as tremor and dystonia however indicated the first benefits for patients [14,15,16]. The underlying mechanisms of DBS are still not sufficiently identified and therefore the extensive current assumptions about DBS functional principles are discussed elsewhere in more detail [17,18].

In short, underlying mechanisms include local and network-wide effects and might even range to neuroprotective and neurogenesis effects, even though evidence is preliminary here. While high frequency stimulation seems to mimic a lesion in the targeted area [19,20], the frequency of the action’s potential output in this certain region increases [18]. Therefore, no one unified mechanism such as the inhibition of neuronal activity can explain the DBS overall effect on the region of interest. A dissociation between the somatic and axonal activity of the neurons may explain these contradicting patterns. As a consequence of different thresholds for somatic and axonal neuronal activity, DBS might inhibit the soma near to the stimulated electrode, but activate axons and dendrites in the stimulated area, which results in an increase of the action potential output frequency [18]. Notwithstanding, these inhibition and activation effects are merely two out of several mechanisms contributing to the impact of DBS.

The overall effect of DBS in the globus pallidus internus (GPi) is beneficial to hyperkinetic movement disorders like dyskinesia in Parkinson’s disease (PD) [21,22], primary dystonia [23,24], tardive dyskinesia [25,26] and other disorders like neurodegeneration with Brain Iron Accumulation [27], chorea-akanthocytosis [28,29] or dystonia–choreoathetosis in cerebral palsy [30,31,32]. As an alternative method to pallidotomy as a treatment option for HD, DBS (especially of the GPI) has been of growing interest during the last 12 years [33,34].

## 3. Invasive Assessment of the Basal Ganglia Network in HD

**Figure 2:**
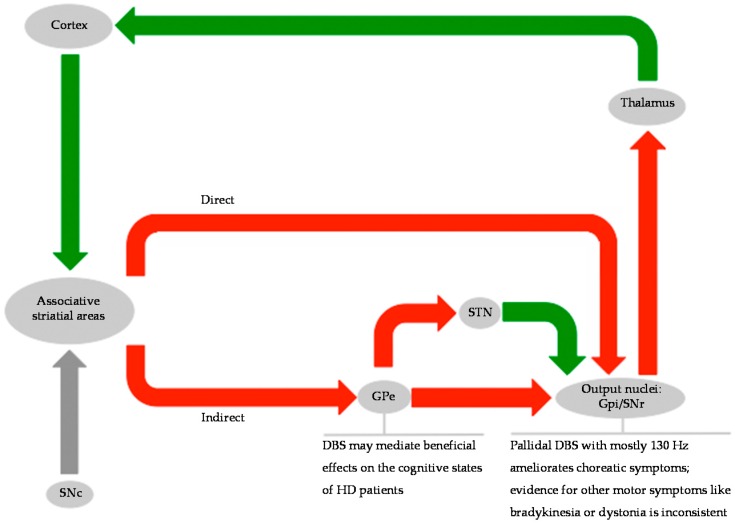
Basal ganglia network and targets for DBS in HD. Red arrows indicate inhibitory, green arrows indicate excitatory connections.

The disruption of the cortico-striato-thalamo-cortical (CSTC) networks is assumed to be the underlying functional mechanism of HD and presumably is linked to cellular degeneration [35]. Three parallel arranged circuits—an associative, motor and limbic circuit—can be distinguished [36]. Due to the evolution of the three systems, a functional segregation of these networks is assumed. Nevertheless, a shared hierarchic CSTC-architecture can be found (see Figure 2): Cortical glutamatergic projections reach the associative striatal areas, from where a direct and an indirect pathway reach the output nuclei of the basal ganglia system [37]. The direct pathway comprises the following circuit: Distinct neurons of the associative striatal areas project via inhibitory (GABA-ergic) transmission to output nuclei of the basal ganglia system [38], which connects again via GABA-ergic projections to certain parts of the thalamus, that eventually indicate glutamatergic efferents to cortical areas. Depending on the certain function of a circuit the involved anatomical structures of striatum, output nuclei, and thalamic nuclei vary, e.g., the motor circuit involves the putamen, GPi and the anterior ventral thalamic nucleus. On the other hand, the indirect pathway comprises different stations: either it solely passes the globus pallidus externus (GPe; GABA-ergic) or the indirect pathway reaches the output nuclei by transversing both the GPe and subthalamic nucleus (STN, glutamatergic) [38]. The loss of striatal neurons, which reach the GPe within the “indirect pathway”, is characteristic and probably pivotal in early stages of HD [39,40]. The consequences are, firstly, the relatively overactive GPe, secondly, the increased inhibition of the STN [41,42], thirdly, the suppression of the output nuclei and, eventually, the disinhibition of thalamic nuclei. Hence, the loss of striatal neurons results in a thalamic overactivity. Choreatic movements derive from the increased thalamic output in the basal ganglia motor loop. In contrast to this, early cognitive impairment, e.g., the inhibition of error control, may arise from the impairment of the associative CSTC circuit [43]. With respect to an affection of the third basal ganglia loop, i.e., the limbic circuit, findings suggest altered affectivity in HD, such as agitation, irritability, anxiety, or euphoria [44]. As HD progresses, alteration of striatal efferents of the direct pathway play a more significant role. Concurrently, hyperkinetic-rigid symptoms aggravate at the expense of initial choreatic symptoms, so that this shift of symptoms could relate to the direct pathway affection [45]. The assumption of open connections between the different circuits is an additional concept to the aforementioned closed loop projections. This concept facilitates interaction at different hierarchical levels of the CSTC network [46] such as directional input from the associative CSTC circuit to both the motor and limbic loops. Findings in histology as well as in morphometry indicate an early affection of the associative CSTC loop. Assuming a (relative) functional integrity of the three main CSTC circuits, the idea of an open connection between those offers an explanation for the motor and limbic symptoms, which manifest subsequently [46].

Prior to the implantation of the DBS electrodes, one method to determine the precise, circumscribed target position is the invasive electrical recording of multi-unit recordings of action potentials. On the other hand, these microelectrode recordings are also useful in the research of dysfunctional electrophysiological processes in e.g., movement disorders [47]. As such, they serve to uncover circumscribed characteristic neuronal patterns. The comparison of invasive electrical recordings without sedation between PD and idiopathic dystonia indicates disease-specific pallidal activation patterns. While the measured average GPe discharge rates for dystonia and for PD are almost identical (~55 Hz), both diseases differ distinctly in the GPi discharge rates (PD ~95 Hz, Dystonia ~55 Hz) [48]. Findings of invasive electrical recordings in HD patients are comparatively scarce up to now. Heterogeneous study conditions and populations (such as anesthesia or disease type) result in divergent neuronal firing patterns [34,49]. In contrast to the first published study of HD GPi firing patterns, which investigates one juvenile HD patient under general sedation [34], later studies also focus on discharge rates in non-anesthetized patients [49,50]. In terms of the discharge rate, the findings are inconsistent: While the neurons in the GP indicate a dorsoventral (GPe to GPi) gradient in their discharge rate in DBS surgery in one awake HD patient (~51 to ~73 Hz) [50]. In two non-anaesthetized patients with severe HD, the firing rate of GPi was almost identical to PD: above 80 Hz [49]. Findings with anesthesia [34,51] indicate slower firing of the GPi, around and below 20 Hz, as the use of sedatives is a decisive factor suppressing the discharge rate.

Another method for the electrophysiological characterization of neural networks is the assessment of oscillatory activity via local field potentials (LFP), which reflect synchronized activity of neural clusters in the vicinity of the recording electrode. The analysis of LFP oscillations s by Starr et al. revealed less synchronized neuronal activity in the surrounding of the electrode for the 2–35 Hz frequency range in resting, non-anesthetized HD patients in contrast to PD patients [50]. In another study of one HD patient without sedation from our own group, LFP recordings indicate dorsoventral gradients in the target area [52]. While approaching the GPi center, the power increases in the alpha-theta range (4–12 Hz). We concluded that this alpha-theta dominance could reflect a general characteristic of unvoluntary movements due to corresponding findings in other diseases such as dystonia, levodopa-induced dyskinesia and Tourette’s syndrome. Furthermore, our group observed an even more evident dorsoventral gradient for the low gamma range (35–45 Hz), which intensified when reaching the GPi ventral border. This dorsoventral gradient was considered as crucial pathophysiology for exaggerated motor drive [52].

## 4. Clinical Implications of DBS in HD

### 4.1. Clinical Implications of DBS on Hyperkinetic and Hypokinetic Symptoms

To date, there is only one prospective randomized, double-blind study on the impact of DBS on HD symptoms [53]. Two HD patients with juvenile onset (Westphal variant) and four HD patients with later onset underwent pallidal DBS. Dystonic and bradykinetic symptoms predominated in the Westphal patients, while chorea symptoms were more pronounced in the HD patients with adult onset. In contrast to the two Westphal HD patients, the four other patients could profit extensively from pallidal (GPi or GPe) DBS, as the choreatic symptoms significantly decreased by 60% compared to symptoms’ baseline within the six month DBS treatment. The 60% reduction in choreatic symptoms derives from the acquisition of the UHDRS chorea subscore (Unified Huntington Disease Rating Scale) at baseline and six months after surgery. Although not significant over group, in three out of four non-Westphal patients, marked improvement of dystonia could be observed. In another study with seven HD patients, the 60% reduction in choreatic symptoms could even been measured by the UHDRS one year after implantation [54]. Table 1 and Table 2 illustrate case reports and series with distinct chorea symptom reduction. A total number of 36 patients are reported. As a meta-analysis, chorea reduction can be estimated at around 56%, whereas improvement of Dystonia (scores available from 20 patients) is minor (1%).

In one patient with four implanted electrodes in the bilateral GPi and STN, solely STN DBS failed to reduce the chorea symptoms [65]. On the other hand, STN DBS could play a major role for hypokinesia, as GPi DBS side effects of increased hypokinetic symptoms could be reduced with additional STN DBS in one HD patient [65]. DBS of the GPi seems to cause these hypokinetic side effects such as gait disturbances [69,70,71] and of more pronounced bradykinesia [54,55,64,67]. As a meta-analysis from available bradykinesia-scores in 17 from 36 HD patients, the impairment by GP-DBS is minor (around 3%).

Thus, the few findings in HD patients with DBS on hyperkinetic symptoms of dystonia and hypokinetic symptoms of bradykinesia do not admit an unambiguous recommendation for the stimulation of the pallidum. Beneficial therapeutic effects of well-established pallidal DBS on primary dystonia cannot be transferred to the impact on dystonic symptoms in HD. The few existing studies suggest pallidal DBS to be beneficial [72] or ineffective [54] or negatively impacting [63] on the dystonic symptoms. These heterogeneous findings are also supported in our prospective trial [53] and the above mentioned meta-analysis. Due to the small amount of case reports here, individual pathophysiology could contribute to those contradicting, inconsistent findings of pallidal DBS on dystonic symptoms of HD. Furthermore, depending on the stimulated area of the pallidum, opposite motor effects are known [21].

In terms of unwanted effects, a lower frequency stimulation of 40 Hz could be superior to a higher frequency stimulation of 130 Hz, as choreatic symptoms ameliorated in the same amount under both stimulation frequencies, but hypokinetic symptoms only became less pronounced under the 40 Hz stimulation in three case reports [52,55,58]. Nevertheless, those preliminary results of only three patients have to be interpreted with caution as the overall findings on the optimal stimulation frequency for minimal side effects are inconsistent: High frequency stimulation of more than 100 Hz does not always lead to a worsening of induced hypokinesia [57,60] and DBS of approximately 40 Hz does not always result in a reduction of those hypokinetic symptoms [67,68]. Along with the optimal stimulation area, the precise, most beneficial stimulation frequency is of particular interest for the clinical treatment. According to the few, preliminary existing findings, chorea tends to be suppressed more with higher frequency stimulation compared to lower frequency stimulation. High frequencies of 130 Hz are mostly applied in treatment studies of HD chorea symptoms and, according to some findings, the benefits even increase when using 180 Hz frequencies [57,67,72].

### 4.2. Clinical Implications of DBS on Non-Motor-Functions

Prior to the HD diagnosis based on motoric symptoms, cognitive abilities can decline. Simultaneously, striking physiological changes such as cerebral atrophy become evident [73]. Various cognitive domains such as processing speed, working memory and attention can be affected and the cognitive impairment is progressive in the course of the HD [74]. Deficits in error feedback control mechanisms are regarded as a key problem for cognitive but also motor malfunctions. The improvement of the early cognitive deficits by DBS would contribute to therapeutic treatment, but also to an understanding of physiological dysfunctional mechanisms, as cognitive conspicuities precede motor symptoms [75,76]. In early HD stages, the striatal neurons projecting to the GPe predominantly degenerate, thus positing a major role of the GPe for the cognitive deficits in HD. As such, Ayalon et al. lesioned different parts of the indirect pathway in rats and their results suggest the GPe in primates as a valuable stimulation area to treat cognitive in addition to motor symptoms [77]. Another study sheds light on the cognitive ability of response inhibition in the first transgenic HD rat model. The primate GPe equivalent in rats was stimulated and effectively improved the deficits in the response inhibition [78]. Findings in humans by our own group might point in the same direction, as pallidal DBS in HD patients with preponderant choreatic symptoms over six months was followed by a stable level in cognitive abilities instead of a progressive decline in cognition. Results were slightly, but not significantly better in the GPe-DBS group than in the GPi-DBS group in terms of cognitive effects. This could suggest that pallidal DBS in HD slows down progressive cognitive decline and keeps cognitive abilities on a stable level to some extent [53]. In a recent DBS imaging study, stimulation of the GPe was highlighted with respect to cognitive networks. Nevertheless, this study lacks cognitive tests in order to validate the imaging data [79]. In another experimental study, GPe-DBS had beneficial effects on cognitive control and, here, behavioral as well as electrophysiological data were collected for identification of cognitive effects. Two patients performed an error monitoring task ON and OFF GPe-stimulation: A flanker paradigm was applied to investigate adaptive behavior in response to committed errors. Error-related-behavioral adaptation was compared via the error-related-negativity (ERN) and the post error slowing in the DBS and control group. In addition to this, general response monitoring was measured via the correct-related negativity (CRN/Nc) amplitude for both groups. The findings suggest that GP-DBS positively impacts both aspects, the adaptive behavior as a response to error processing and also the general response monitoring. Smaller ERN, less pronounced post-error-slowing and less pronounced Nc could be observed in manifest HD patients OFF DBS, but their behavioral and electrophysiological measures aligned with the healthy control group when GPe DBS was applied [80]. These are promising findings, which highlight the GPe as a valuable DBS target and suggest cognitive benefits. However, it has to be noted that up to date no placebo-controlled prospective clinical data on GPe-DBS is available. On the other hand, DBS stimulation of the GPi led to far more inconsistent effects, up to now. The effects of GPi-DBS on patients cognition range from a progressive decline similar to non-stimulated HD patients [58,61,67] to stable cognitive functions for at least 4 years [59] and even to alleviation in distinct cognitive abilities [68,62]. Various causes have been discussed for the numerous observed effects of GPi-DBS. According to animal-based findings and studies with humans, it is suggested that GPi-DBS treatment benefits on cognition could derive from electric fields in the GPi, which extend to the GPe. Evidence on other non-motor functions and quality-of life (QoL) is sparse up to date. Existing data from the prospective protocol might suggest some improvement of sub-scales of QoL and depression [70].

## 5. Safety of DBS in HD

In our executed pilot study, the implantation of the DBS electrodes into the GP proved to be a safe procedure and lacked procedure-related side effects. However, these preliminary data have to be treated with caution as they included only six HD patients [53]. Nevertheless, this pilot study is the only one available up to date with a prospective design, which corresponds to the CONSORT criteria with adverse events (AE) entirely reported by using an independent data and safety monitoring board (DSMB). Besides the side effects described in Section 4.1 and Section 4.2, here we focus on the formal safety report of the prospective trial. One might anticipate that DBS causes three main types of adverse device effects (ADE): (1) transient due to electrical stimulation; (2) transient due to technical problems/complication/infections and, finally, (3) transient or permanent due to implantation complications. Concerning all types of AE including ADE, the data from our pilot trial showed the following: AEs that where actually reported within 6 months: eight adverse events were recorded. All AE resolved without sequelae. AEs unrelated to stimulation but possibly due to hospitalization: thrombophlebitis, MRSA nose infection, superficial nose abrasion. AEs related to treatment—thus ADE—were: possibly related to stimulation (Type 1 ADE, exclusively reported with GPi- but not GPe- stimulation): bradykinesia, hyperthermia, gait impairment, increased chorea and possibly related to stimulation system: deactivation of impulse generator (Type 2 ADE). In addition, two serious adverse events (SAE) were reported: gait impairment and hyperkinesia after reprogramming (SAE criterion: leading to hospital admission and requiring reprogramming) and postoperative malignant hyperthermia possibly related to stimulation (SAE criterion: life-threatening and leading to prolonged hospital stay). Both SAE were judged as SADE (Serious Adverse Device Effects) with Type 1. No procedure-related complication or bleeding occurred (Type 3 ADE). In the prospective trial, no side effects on cognition and mood were present.

## 6. Outlook

**Figure 3:**
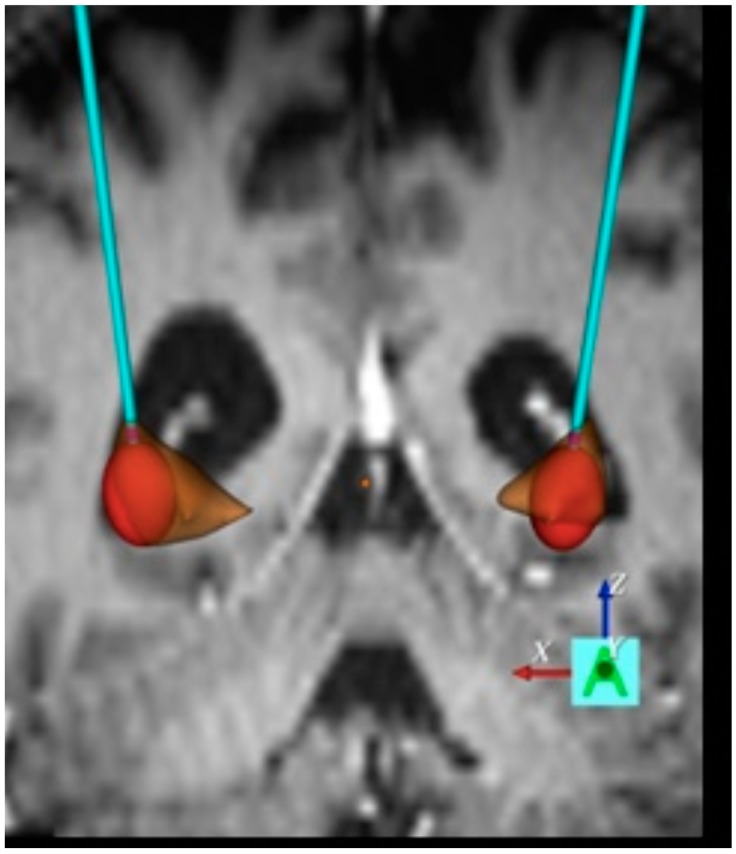
Stimulated target. Example visualization on 3D coronary MRI-view of individual electrodes and volume of tissue activated (VTA, in red) in relation to the pallidum (in brown). Image source: authors’ own contribution.

Preliminary findings in HD patients reveal overall positive effects of pallidal stimulation on chorea. Beside the motor effect on chorea by GPi-stimulation, the presumably better effect-side-effect ratio and the promising findings of GPe-DBS for cognition ought to be further validated. The GPi/GPe border zone might be a suitable target for DBS. One evident difficulty is the progressive atrophy of the GP which might prevent the precise identification of distinct pallidal parts. On the other hand, the atrophic altered GP might lead to the unintended impairment of areas in the surroundings of the target site and thereby provoke unwanted side effects as a consequence of DBS surgery. To overcome these aspects, technical advanced stimulation programming can be used. To identify an optimal treatment of motor symptoms, a systematic investigation of the stimulation frequency is needed, as chorea and bradykinesia treatments were shown to have different, opposing optimal stimulation frequencies. Another further step ought to systemically study the DBS pulse width. As a standard, 60–450 μs were implemented in most cases and, up to now, not much attention has been paid to variations of the pulse width [59,60]. However, an optimal pulse width could warrant larger therapeutic windows and might avoid side effects, as revealed by studies of STN DBS in parkinsonism, in which 30 μs was beneficial [81,82]. Furthermore, the newest DBS devices allow new possibilities concerning pulse width, current steering and directional stimulation [83,84]. The most beneficial treatment approach of direct DBS in HD might be attained by identifying the optimal parameters corresponding to the predominating symptoms in each individual. Optimal stimulation programs could also be achieved by algorithms and models taking into account the volume of tissue active (VTA) and tailored parameters automatically based on anticipated side effects (see Figure 3).

Moreover, sensing neurostimulators will be valuable devices in therapeutic treatment and research. However, contrary to other movement disorders [85], as stated in Section 3, LFP recording data as a possible biomarker in HD is sparse up to date.

In order to create a higher level of evidence for DBS in HD, the next major step is a prospective, randomized, double blind, parallel group, sham-controlled, multi-center (MC) superiority trial which is currently recruiting in Europe (ClinicalTrials.gov: NCT02535884). Based on the evidence outlined in this review article, the ongoing MC-randomized controlled trial is focusing on the efficacy of GP-DBS on chorea as a primary endpoint while considering several motor functions such as dystonia and bradykinesia, cognition, mood and quality of life as secondary endpoints. Patients with predominant chorea despite best medical treatment (UHDRS chorea sub score ≥ 10) with only minor cognitive and psychiatric disturbances are selected. Postural instability is considered as exclusion criteria for DBS. In terms of risk management, based on the preliminary data, the HD cohort does not seem to be at special risk due to DBS when all inclusion/exclusion criteria of patients are carefully addressed. This assumption is based on the data with three Type 1 and one Type 2 ADE and no Type 3 ADE in the pilot data [70]. For the most serious Type 3 complications, we propose the following risk stratification: Risk of brain bleeding is stratified with grade of brain atrophy:

(1) No significant atrophy;
(2) Mild cortical atrophy as common in neurodegenerative disorders;
(3) Severe cortical atrophy and additional atrophy periventricular and of the target basal ganglia structures.

Grade 1 atrophy is not expected in neurodegenerative diseases such as Parkinson’s and Huntington’s disease. Grade 2 is common in these diseases and results in a risk of bleeding of (5%–7%) during implantation [86]. As Grade 3 atrophy makes a surgical approach more difficult due to the atrophy of the target area, it is assumed that these patients have higher operative risks. However, no systematic data on these patients is available. Grade 3 atrophy is more common in HD patients at moderate stages than in PD patients. Thus, these patients are excluded in order to keep the implantation risk at the level of PD of 5%–7%. Thus, due to the mentioned risk assessment and calculation, it is assumed that the implantation risk in HD patients with brain atrophy Grade 2 is at the level of already approved and CE marked indication of DBS.

## 7. Conclusions

There is preliminary evidence for the usefulness of pallidal DBS for chorea suppression in HD from a number of cases, case series and smaller trials (with fewer than 10 patients per trial) and from one prospective randomized, double-blinded trial lacking a placebo control group. DBS procedure was demonstrated to be a safe treatment option in the above mentioned trial. Cognitive functions might benefit from stimulation of the external part of the pallidum. Up to date, DBS effects on chorea and other motor symptoms such as dystonia and on QoL are examined in a larger and placebo (OFF-stimulation) controlled trial.

## Figures and Tables

**Table 1:** Case reports (*n* = number of patients) of DBS in HD.

Study	*n*	FUP (Months)	Age (Years)	DisDur (Years)	Chorea	Bradykinesia	Dystonia	Total Motor	Comments
Moro et al. [55]	1	8	43	8	44%	14%	38%	31%	DBS frequency of 130 Hz but not 40 Hz worsened bradykinesia. Increased regional cerebral blood flow in cortical motor regions.
Fawcett et al. [56]	1	4	42	n.a.	56%	n.a.	60%	26%	Moderate improvement of speech, swallowing and gait, task-specific improvement of oculomotor function.
Hebb et al. [57]	1	12	41	13	57%	n.a.	n.a.	15%	Chorea improves with higher stimulation frequency (180 Hz), no frequency-dependent effect of bradykinesia
Fasano et al. [58]	1	12	72	17	77%	60%	100%	n.a.	Worsening of gait, apathy, cognitive decline, functional gain minimal, turning off at 11 months did not induce chorea
Biolsi et al. [59]	1	48	60	10	21%	n.a.	n.a.	5%	Chorea reduced by 56%, when comparing DBS-on vs. DBS-off. L-Dopa-responsive worsening of bradykinesia. Cognition stable.
Groiss et al. [52]	1	12	65	n. a.	47%	n.a.	31%	n.a	Primary focus on local field potentials. Hypokinesia observed at 180 Hz stimulation improved from 40 Hz DBS
Garcia-Ruiz et al. [60]	1	12	30	10	n.a.	n.a.	n.a.	48%	Marked improvement of vocalization. No effect of DBS on hypokinesia and rigidity. Facilitated activities of daily living.
Spielberger et al. [61]	1	48	30	9	75%	5%	70%	−4%	Worsening of chorea with 40 Hz DBS, best results with 130 Hz DBS. Progression of bradykinesia compatible with natural course.
Huys et al. [62]	1	12	40	3				16%	Improved cognition 6 months after surgery followed by a decline at 12 months (but improved results compared to baseline assessment)
Velez-Lago et al. [63]	1	60	60	2	56%	n.a.	−40%	−98%	Chorea (69%), dystonia (40%), and overall motor score (37%) improved up to 24 months after surgery. Stable cognition.
Cislaghi et al. [64]	1	48	31	16	67%	n.a.	n.a.	n.a.	Significant improvement in chorea in juvenile HD. Impairment of bradykinesia. No effect on cognitive function.
Gruber et al. [65]	1	48	41	9	60%	42%	50%	19%	GPI DBS-induced bradykinesia alleviated with STN DBS. Cognitive decline compatible with the natural course of HD.
Loutfi et al. [66]	1	12	59	12	27%	n.a.	−40%	12%	Stable cognition, modest improvement of verbal fluency, marked improvement of behavioral assessment.

Improvement is indicated as percentage of baseline scores (negative values indicate impairment). FUP = Follow-up period. DisDur = disease duration. Table adapted from [1].

**Table 2:** Case series and trials (*n* = number of patients) of pallidal DBS in HD.

Study	*n*	FUP (Months)	Age (Years)	DisDur (Years)	Chorea	Bradykinesia	Dystonia	Total Motor	Comments
Kang et al. [67]	2	24	57	10	63%	−11%	n.a.	22%	Best results on chorea with either 40 Hz (patient 1) or 130 Hz (patient 2) DBS. Cognitive decline compatible with natural disease progression.
		24	50	5	59%	0%	0 to 6	4%	
Velez-Lago et al. [63]	2	12	27	7	74%	n.a.	n.a.	43%	Despite good effect on chorea, there was no improvement for a patient with predominant dystonia. Worsening of bradykinesia and rigidity
		9	19	6	n.a.	n.a.	20%	−24%	
Gonzales et al. [54]	7	36	78	5	65%	n.a.	n.a.	−10%	Bradykinesia worsened over time. Additionally, DBS-dependent effects could be observed. Reduction of pulse width reduced bradykinesia. Non-significant worsening of dystonia over time. Despite progressive decline of cognition, cognitive levels were not significantly worse compared to baseline
		36	39	8	69%			14%	
		36	74	4	20%			−11%	
		36	54	8	79%			−30%	
		36	37	3	67%			33%	
		12	30	3	70%			40%	
		12	36	3	80%			−64%	
Wojtecki et al. [53]	6	6	52	3	66%	5%	56%	42%	First randomized, double-blind study up to date. First study comparing GPE and GPI DBS. Patients 4 and 5 suffered from juvenile variant of HD and therefore exclusively presented with hypokinetic-rigid symptoms and dystonia. DBS of GPE and GPI did not lead to significantly different results. Heterogeneous results concerning functional outcome.
		6	71	21	63%	−9%	85%	27%	
		6	38	10	46%	−22%	55%	11%	
		6	25	11	n. a.	−19%	−44%	−3%	
		6	23	8	n. a.	−10%	−37%	−9%	
		6	29	4	66%	17%	0%	28%	
Zittel et al. [68]	3	36	54	5	50%	−36%	100%	11%	Heterogeneous results concerning DBS effects on bradykinesia and dystonia. Mini-mental status examination stable over time, while more complex tests revealed diverging results
		12	35	4	58%	11%	−250%	25%	
		12	45	7	40%	39%	−100%	20%	
Delorme et al. [51]	3	30	56	10	15%	−100%	0 to 1	−18%	Greater effect size (with mean improvement of 55% of chorea and 32% for the total score), if not compared to baseline but DBS off assessment at follow-up. DBS via ventral electrode contacts was more effective than DBS via dorsal contacts
		24	24	3	67%	0 to 4	0 to 6	20%	
		12	50	10	29%	−33%	−140%	−2%	

Improvement is indicated as percentage of baseline scores (negative values indicate impairment). If a percentage could not be calculated, since the initial value was 0, raw data are provided. FUP = Follow-up period. DisDur = disease duration. Table adapted from [1].
